# Tonsillar Actinomycosis Associated With Cervical Lymphadenopathy: A Rare Benign Condition Mimicking Malignancy

**DOI:** 10.7759/cureus.107556

**Published:** 2026-04-22

**Authors:** Sara Elaissaoui, Hicham Mimouni, Rajae Borki, Ilham Rkain

**Affiliations:** 1 Department of Otorhinolaryngology-Head and Neck Surgery, Mohammed VI University Hospital - Tangier, Tangier, MAR; 2 Department of Otorhinolaryngology, Faculty of Medicine and Pharmacy of Tangier, Tangier, MAR; 3 Department of Anatomy, Faculty of Medicine and Pharmacy of Tangier, Tangier, MAR

**Keywords:** case report, chronic tonsillar granulomatous infection, oropharyngeal actinomycosis, tonsillar actinomycosis, tonsillar asymmetry, tonsillar carcinoma mimicry

## Abstract

Tonsillar actinomycosis is an uncommon chronic infection that can clinically and radiologically mimic malignancy. Because of its nonspecific presentation and tumor-like appearance, particularly when associated with cervical lymphadenopathy, it often poses a diagnostic challenge. A 20-year-old patient presented with a four-month history of progressive left tonsillar hypertrophy. Nasofibroscopy showed an irregular tonsillar surface with nasopharyngeal bulging, raising suspicion of a neoplastic lesion. Contrast-enhanced CT revealed asymmetric left tonsillar enlargement with homogeneous thickening of the nasopharyngeal wall and suspicious contralateral lymph nodes. Laboratory findings were within normal limits. The patient underwent left tonsillectomy with adenoidectomy. Histopathological examination demonstrated tonsillar hyperplasia containing Actinomyces colonies and adenoidal lymphoid hyperplasia, without evidence of malignancy. The patient received a six-week course of oral amoxicillin, resulting in complete regression of the cervical lymphadenopathy and no recurrence during follow-up.This case underlines the importance of considering tonsillar actinomycosis in the differential diagnosis of unilateral tonsillar hypertrophy mimicking malignancy, especially when accompanied by cervical lymphadenopathy. Early histopathological diagnosis and combined surgical and antibiotic therapy ensure optimal outcomes while avoiding unnecessary radical procedures.

## Introduction

Actinomycosis is a rare, chronic bacterial infection caused by Actinomyces species, which are part of the normal oral flora. Although cervicofacial actinomycosis is relatively well recognized, primary involvement of the tonsils is exceedingly rare. It is a rare infectious disease with slowly progressive, chronic suppurative lesions, often mistaken for malignancies due to its ability to mimic them [1]. Clinically, tonsillar actinomycosis may manifest as unilateral tonsillar enlargement or chronic tonsillitis, occasionally mimicking malignancy due to its atypical appearance and associated lymphadenopathy. The definitive diagnosis relies on histopathological examination, while optimal management consists of surgical excision followed by a prolonged course of antibiotic therapy [2]. Prompt recognition is crucial to prevent unnecessary invasive procedures and to guide effective management. Here, we present a rare case of tonsillar actinomycosis in a young adult, highlighting its clinical, radiological, histopathological, and therapeutic features. This case report has been reported in line with the Surgical CAse REport (SCARE) 2025 checklist [3].

## Case presentation

**Figure 1:**
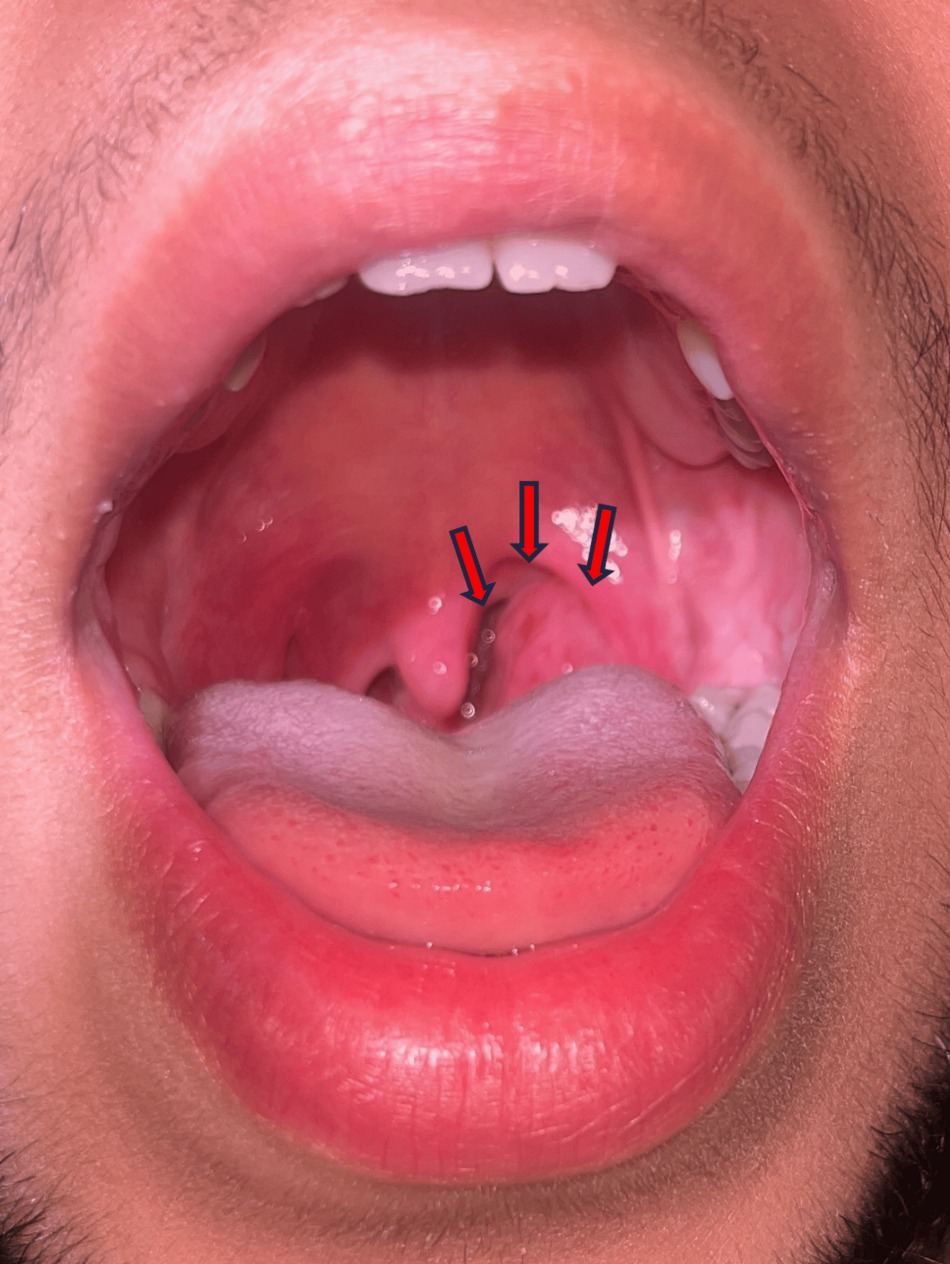
Enlarged left tonsil (arrows) with a cryptic surface

A 20-year-old patient with no significant past medical history presented with a four-month history of progressive left tonsillar hypertrophy associated with intermittent odynophagia but no fever, weight loss, or constitutional symptoms. Clinical examination revealed a markedly enlarged left tonsil with a cryptic surface, nearly crossing the midline and partially obstructing the oropharyngeal airway (Figure 1).

The right tonsil appeared normal. Palpation of the neck showed an ipsilateral, mobile, non-tender cervical lymph node of about 2 cm, with no overlying skin inflammation or fixation to surrounding tissue, in the upper jugular region.

**Figure 2:**
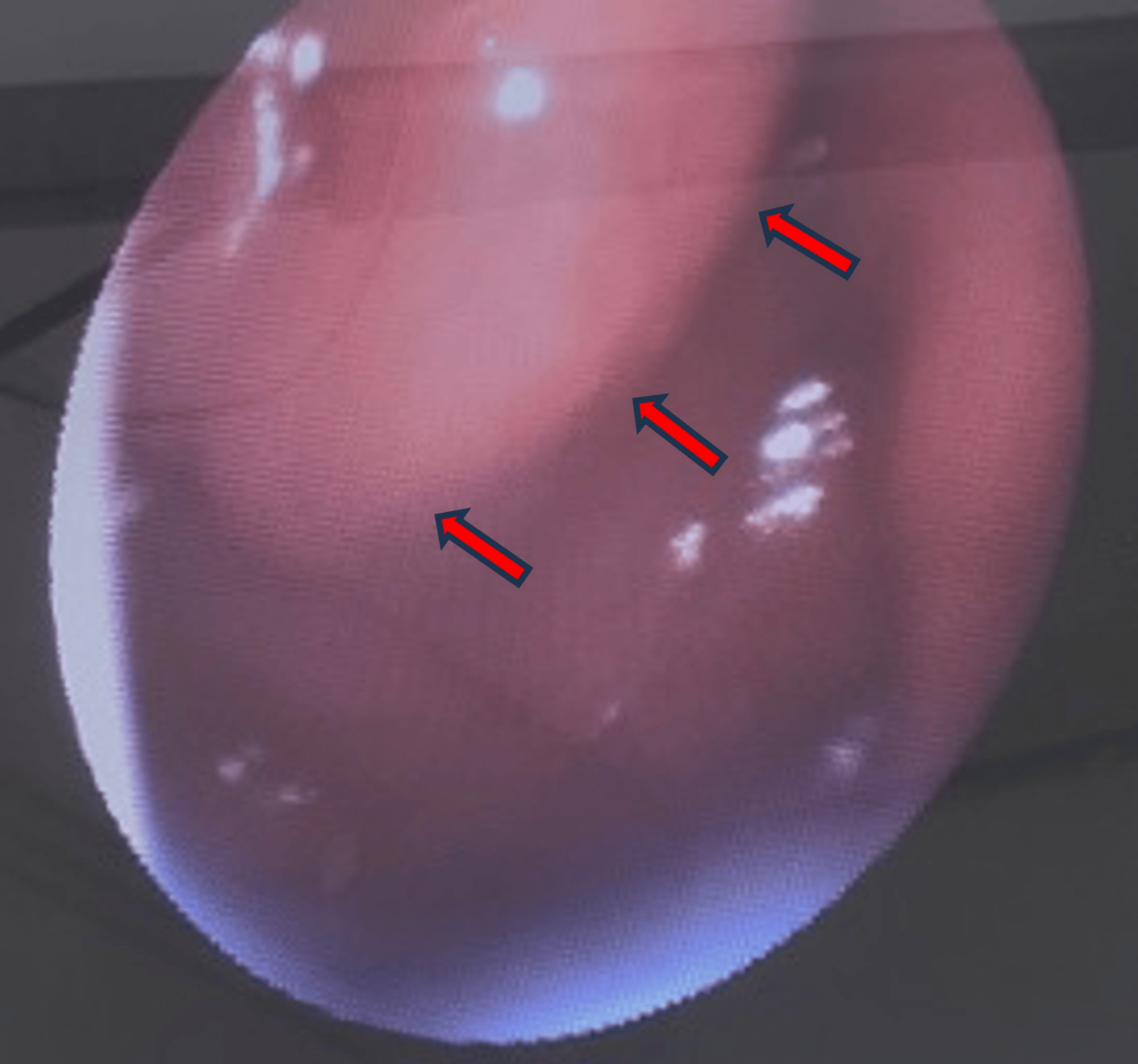
Nasopharyngoscopic view showing an irregular nasopharyngeal bulge (arrows)

Nasofibroscopic evaluation demonstrated an irregular nasopharyngeal bulge with left tonsillar hypertrophy, suggestive of a possible neoplastic process (Figure 2).

Routine hematological and biochemical tests were within normal limits.

**Figure 3:**
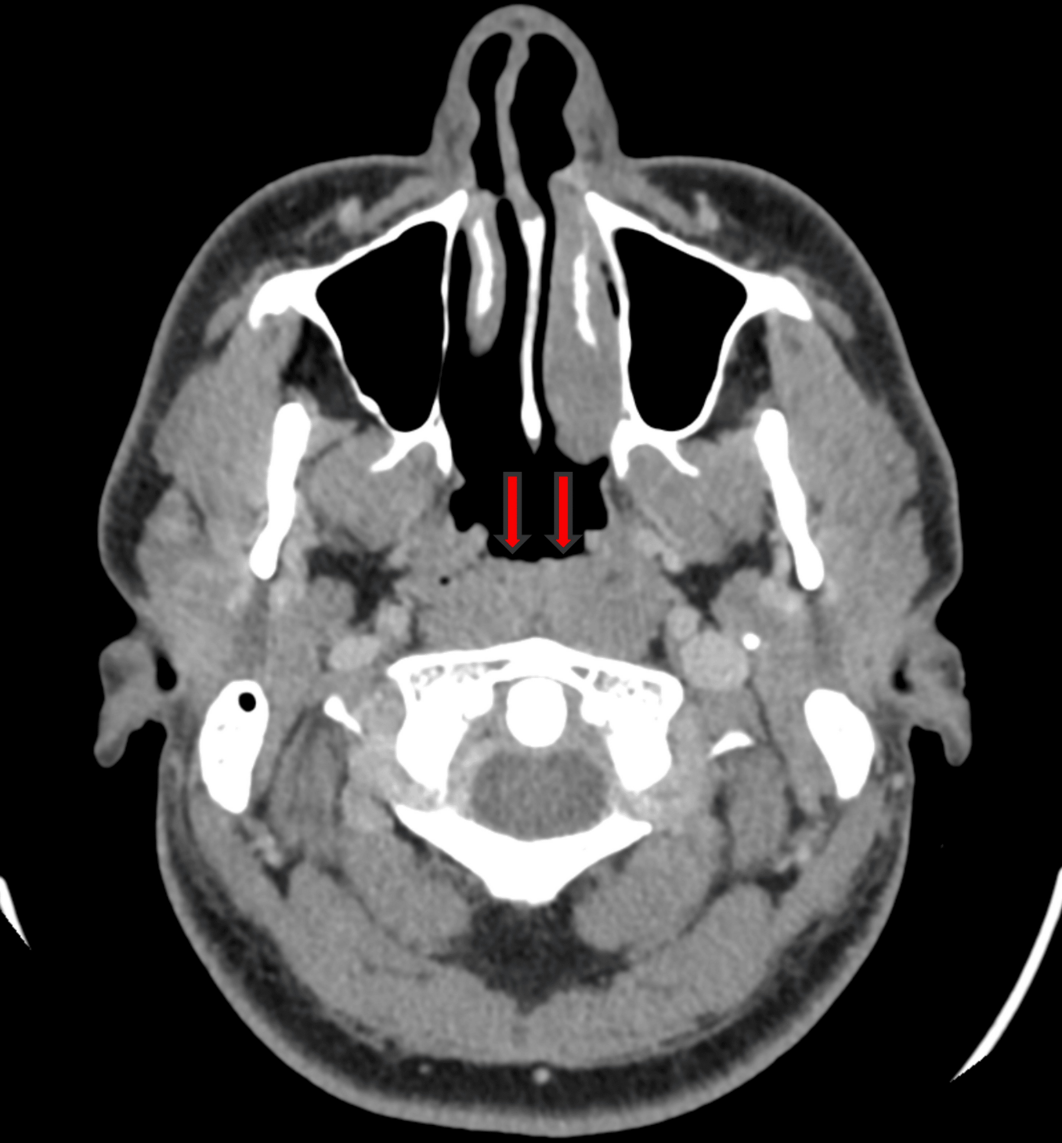
Axial CT image showing homogeneous thickening of the posterior-superior nasopharyngeal walls (arrows)

CT imaging demonstrated homogeneous thickening of the posterior-superior nasopharyngeal walls, measuring up to 16 mm at its maximum thickness, with regular contrast enhancement (Figure 3).

**Figure 4:**
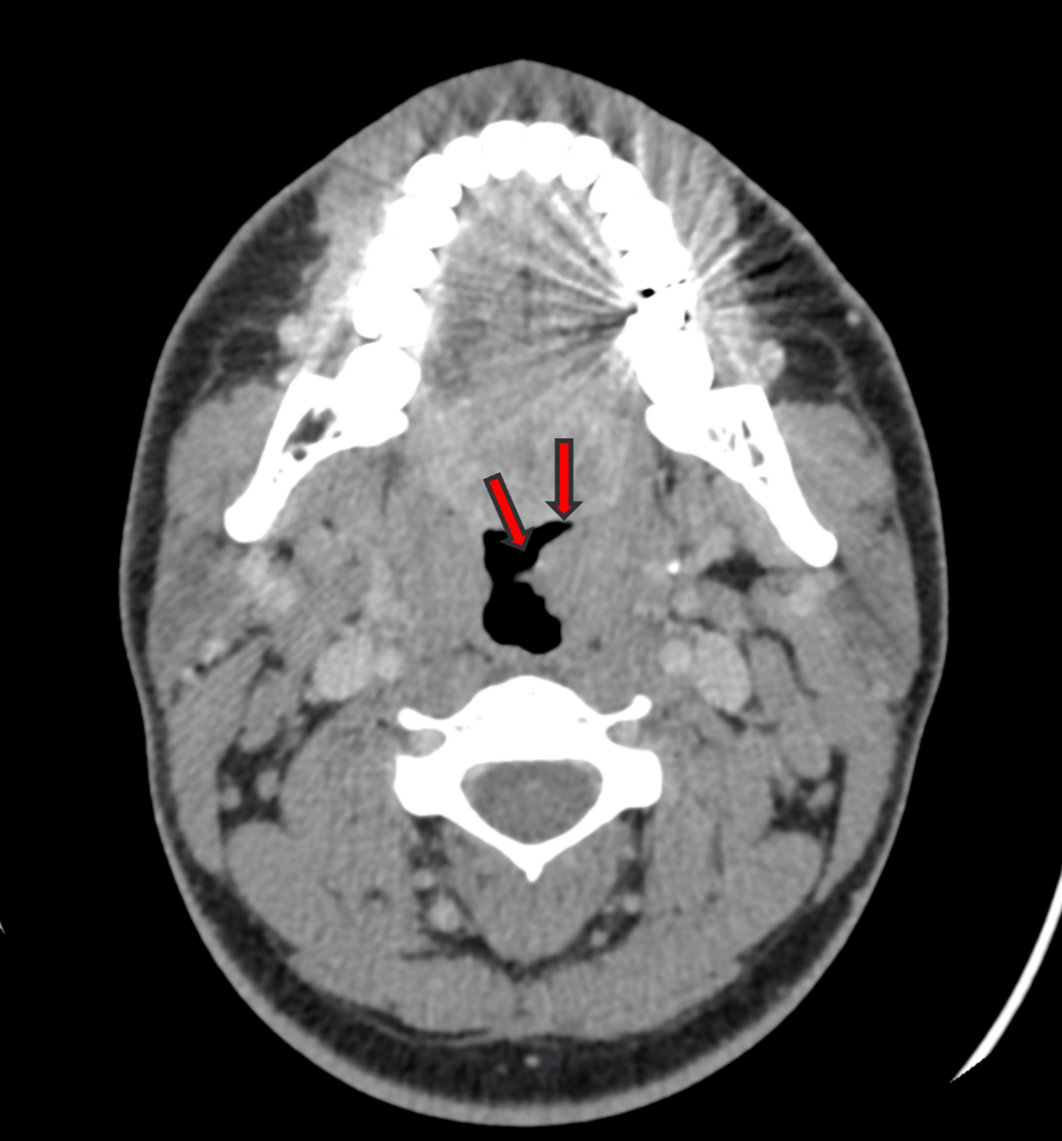
Axial CT image showing left palatine tonsillar hypertrophy (arrows)

The nasopharyngeal cavity appeared to bulge slightly. Bilateral palatine tonsillar hypertrophy was observed, more pronounced on the left, causing partial narrowing of the oropharyngeal lumen (Figure 4).

**Figure 5:**
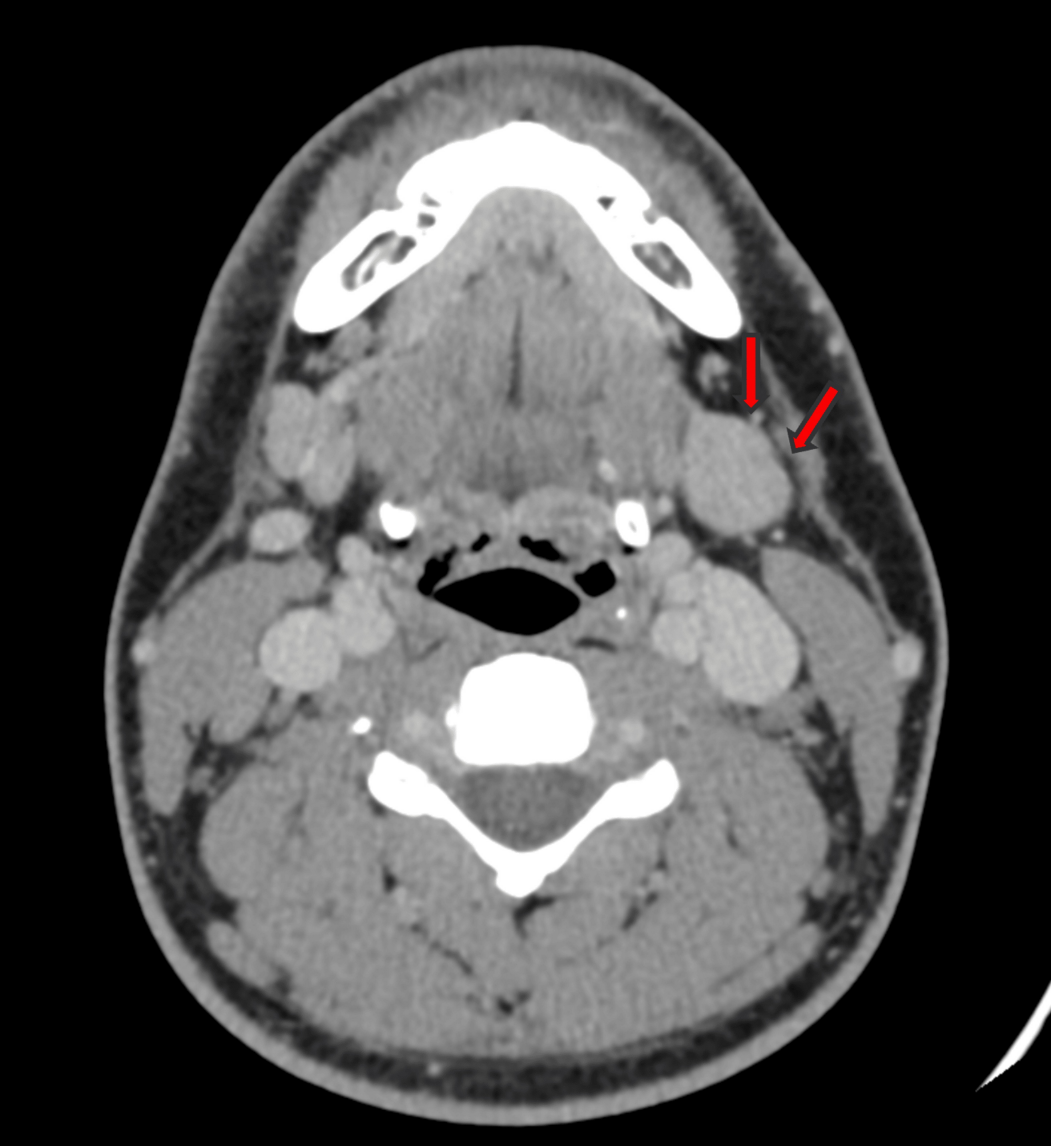
Axial CT image showing right submandibular cervical lymph node with an irregular contour suggestive of a suspicious lesion (arrows)

Mild mucosal thickening of the inferior turbinates was also noted. A right submandibular cervical lymph node with an irregular contour was identified in the posterior cervical space, measuring 10 mm in short-axis diameter, suggestive of a suspicious lesion (Figure 5).

The patient underwent left tonsillectomy combined with adenoidectomy under general anesthesia. The postoperative course was uneventful. 

**Figure 6:**
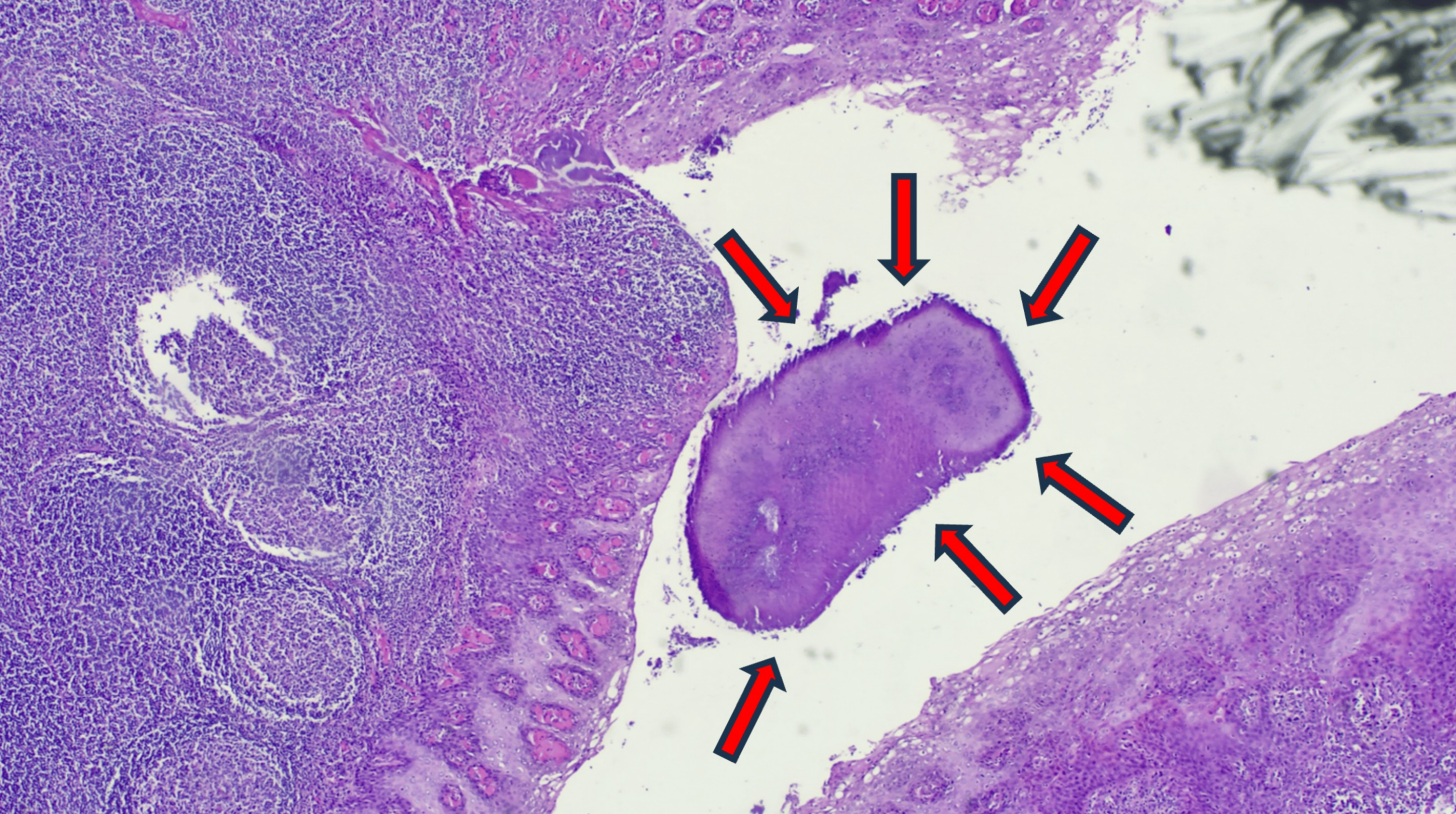
Histopathological section showing clusters and scattered granules consistent with Actinomyces colonies (arrows)

Histological examination revealed in the tonsillar resected specimen lymphoid parenchyma delineated by fibrofatty tissue. The tissue contained lymphoid follicles of variable size, each displaying a germinal center with a light zone rich in immunoblasts and a broader secondary dark zone, surrounded by a mantle of lymphocytes. Clusters and scattered granules consistent with Actinomyces colonies were identified (Figure 6), surrounded by a chronic inflammatory reaction.

No signs of dysplasia or malignancy were observed. The adenoid tissue showed reactive lymphoid hyperplasia.

Following surgery, the patient received oral amoxicillin for six weeks (3 g per day). During follow-up, the previously noted lymphadenopathy regressed completely, and no additional cervical surgery was necessary. The patient recovered fully without complications and with no evidence of recurrence. 

## Discussion

Actinomycosis is a chronic suppurative infection that may develop in almost any part of the body. The cervicofacial form represents the most common presentation and typically arises when bacteria penetrate through a disrupted mucosal barrier, most frequently of odontogenic origin [4]. Historically, actinomycosis was first identified over a century ago as a chronic, suppurative, and granulomatous bacterial disease [5]. The earliest description, reported by von Langenbeck in 1845, initially misclassified the organism as a fungus. Later, in 1877, Harz introduced the term Actinomyces, meaning “ray fungus.” Subsequent investigations, however, confirmed that the pathogen is a Gram-positive, non-spore-forming, anaerobic or microaerophilic bacillus. Among the different species, Actinomyces israelii remains the most frequent etiological agent in humans, although polymicrobial co-infections are common [6].

Members of the genus Actinomyces are Gram-positive anaerobes that normally inhabit the oral cavity as part of the resident human microbiota. They are occasionally implicated in localized infections, particularly when mucosal integrity is compromised. Although tonsillar colonization by Actinomyces has been described in several studies, its relationship with clinical presentation, surgical indications, and postoperative outcomes remains uncertain [7]. Tonsillar actinomycosis is an uncommon condition, despite the fact that its causative organisms are regular commensals of the oropharynx. These bacteria may colonize the tonsillar crypts, leading to infection even in otherwise healthy individuals [8]. The composition of the oral microbiome is influenced by multiple host and environmental factors, including dietary habits, body mass index, and smoking. Interestingly, Actinomyces species appear to be more prevalent in the palatine tonsils of overweight or obese patients [9].

Clinically, tonsillar actinomycosis presents as a chronic and nonspecific disease, and in some instances, it may manifest atypically as unilateral tonsillar hypertrophy [2]. The most common differential diagnoses for asymmetric tonsillar enlargement are squamous cell carcinoma and lymphoma. However, tonsillar actinomycosis should always be considered as a rare but possible cause [4]. In this context, Carvalho de Medeiros et al. reported a case of tonsillar actinomycosis that closely mimicked a relapse of non-Hodgkin lymphoma [5].

In general, tonsillar actinomycosis is a rare, chronic infection affecting the tonsillar tissue secondary to invasion by anaerobic bacteria from the oral cavity. It frequently presents as tonsillar hyperplasia and may simulate malignancy [2]. In 2024, Montenegro et al. published a case emphasizing the importance of a multidisciplinary approach in the diagnosis and management of actinomycosis, as the disease can resemble malignant or granulomatous conditions such as tuberculosis [1]. According to Samaila et al., there are no pathognomonic symptoms that enable clinical diagnosis of tonsillar actinomycosis, apart from obstructive manifestations such as dyspnea, snoring, and sleep apnea, which were also observed in our patient. Tonsillar enlargement often coexists with chronic or recurrent tonsillitis, thus prompting tonsillectomy-especially in individuals with Grade three or four tonsillar hypertrophy, which is more likely to cause obstructive sleep apnea or recurrent infections, as was the case in our patient cohort. Diagnosis is often incidental on histopathological examination of tonsillectomy specimens obtained for various indications. In their series, patients received parenteral high-dose penicillin for four weeks, followed by four months of oral therapy [8]. In contrast, in our case, the infection was localized, and surgical excision of both tonsils and adenoid tissue, combined with a six-week course of oral amoxicillin, proved sufficient for complete resolution.

Although tonsillar actinomycosis is generally considered a benign and non-alarming infection, Péziers et al. reported a rare and dramatic presentation in elderly, immunocompromised patient who experienced a herald bleed from a tonsillar lesion. Despite prompt surgical control, the patient succumbed to a carotid blowout, and autopsy findings revealed invasive actinomycosis without any malignancy [10]. 

Finally, the presence of Actinomyces colonization in tonsillar tissue may contribute to chronic hypertrophy and long-term tonsillar pathology such as chronic tonsillitis refractory to antibiotics. Nevertheless, there appears to be no consistent correlation between such colonization and the usual indications for tonsillectomy, including recurrent tonsillitis or sleep-disordered breathing [11].

## Conclusions

Actinomycosis remains a diagnostic challenge due to its ability to mimic neoplastic or granulomatous diseases. To the best of our knowledge, no previously published case has described a concomitant presentation of tonsillar actinomycosis with associated cervical lymphadenopathy. Our case highlights an uncommon presentation of tonsillar actinomycosis associated with cervical lymphadenopathy, closely mimicking a neoplastic process. Awareness of this rare association is crucial for accurate differential diagnosis, especially in patients presenting with unilateral tonsillar hypertrophy and cervical masses. Early histopathological confirmation and a combination of surgical excision and targeted antibiotic therapy remain essential to ensure complete recovery and to avoid unnecessary oncologic procedures. From a practical perspective, tonsillar actinomycosis should be considered in the differential diagnosis of unilateral tonsillar enlargement with suspicious clinical or imaging features. In such cases, histopathological examination remains essential, and complete excision may be preferred over biopsy when malignancy cannot be excluded.
